# The associations of different recess types on physical activity and sedentary behaviour in Estonian primary school students

**DOI:** 10.1093/eurpub/ckaf052

**Published:** 2025-04-07

**Authors:** Getter Marie Lemberg, Merike Kull, Jarek Mäestu, Eva-Maria Riso, Evelin Mäestu

**Affiliations:** Institute of Sport Sciences and Physiotherapy, Faculty of Medicine, University of Tartu, Tartu, Estonia; Institute of Sport Sciences and Physiotherapy, Faculty of Medicine, University of Tartu, Tartu, Estonia; Institute of Sport Sciences and Physiotherapy, Faculty of Medicine, University of Tartu, Tartu, Estonia; Institute of Sport Sciences and Physiotherapy, Faculty of Medicine, University of Tartu, Tartu, Estonia; Institute of Sport Sciences and Physiotherapy, Faculty of Medicine, University of Tartu, Tartu, Estonia

## Abstract

Students’ physical activity (PA) levels tend to decrease with increasing age; however, the school day structure can potentially influence students’ PA levels. This study aimed to measure and compare objective levels of PA during recess and school time between schools with different recess types. 15 different schools were selected, and accelerometry-based PA levels of 9–13-year-old students were measured. Schools were selected based on the recess types: (i) ‘daily outdoor recess’; (ii) ‘irregular outdoor recess’; (iii) ‘indoor recess’. The ‘daily outdoor recess’ group reached the highest moderate-to-vigorous PA (MVPA) during recess compared to other groups. ‘Indoor recess’ group had more sedentary time during recess compared to ‘irregular outdoor recess’ and ‘daily outdoor recess’ groups (43.6 ± 1.0%, 34.0 ± 1.0%, 30.8 ± 0.8%, respectively; *P* < .05). Time in MVPA was unchanged during recess across all grades in the ‘daily outdoor recess’ group (from 22.8% to 25.6%), while decreased MVPA from 27% to 17% and from 21% to 10% was found in ‘irregular outdoor recess’ and ‘indoor recess’ group, respectively; *P* < .05. Sedentary time increased from 34% to 52% in the ‘indoor recess’ group and from 26% to 43% in the ‘irregular outdoor recess’ group (*P* < .05). There was a positive association between the recess length and MVPA minutes acquired during recess; however, during a 15–25 min outdoor recess, students spent more time in MVPA compared to a 30–50 min outdoor recess (28%–30.5%, 21%–23%, respectively). The findings emphasize that unstructured outdoor recess has a high potential to maintain the MVPA levels with increasing age.

## Introduction

Insufficient physical activity (PA) levels are one of the main causes of various health problems for children, including overweight, obesity, and cardiorespiratory diseases [1]. In Estonia, only 43% of youth (7–17-year-olds) meet the global PA recommendation of the average of 60 min of moderate-to-vigorous PA (MVPA) per day [2]. Across the lifespan PA decreases, and sedentary time increases with age [3]. Therefore, World Health Organization (WHO) stresses both increasing PA levels and limiting sedentary behaviour among children and adolescents [1]. Thus, developing and investigating PA interventions for children and adolescents is essential.

Schools are powerful agents for PA interventions [4, 5], because students spend most of their waking hours at school, and schools can reach children from various socioeconomic backgrounds. Recess, especially outdoor recess, has been mentioned as the most beneficial part of the school day contributing to the increase of MVPA levels [3, 6]. Recess should be an unstructured outdoor break facilitating various activities, including play, socialization, and reflection [7]. Additionally, unstructured outdoor play is essential for cognitive restoration and improved focus [7, 8].

PA levels of indoor and outdoor recess have been previously compared, and outdoor recess is known to boost PA [9–11], but the influence of diverse recess types and durations on MVPA is unstudied. A systematic review found that students can acquire even more MVPA during recess compared to physical education lessons [3]. While there is no definitive recommendation for optimal recess duration to maximize MVPA accumulation, few authors suggest spending 40%–50% of recess time in MVPA [6, 12, 13]. whereas Erwin *et al*. found that even a 15 min recess can contribute significantly to students’ school time PA [6]. However, the outdoor recess alone does not ensure increased student PA, the design of the schoolyard plays also a crucial role [11]. Diverse schoolyards, featuring varied play structures, open spaces, and natural elements, promote more active engagement during recess [11, 13]. Beyond increasing PA levels, outdoor recess and well-equipped schoolyards are essential for motor development and physical literacy [14]. Versatile schoolyard facilities promote the acquisition of fundamental skills like balancing and throwing etc. [11, 15]. highlighting the value of free play during the school day.

In Scandinavian countries like Norway and Finland outdoor recess has been a common part of the school day already for years [16, 17], whereas in Estonia, outdoor recess is a fairly new addition to the school day and recess is mainly spent indoors [11]. The Schools in Motion program is an educational innovation program in Estonia that focuses on increasing students’ PA levels by promoting various PA opportunities throughout the school day [5]. Outdoor recess is one of many PA possibilities that the Schools in Motion program recommends to their member schools. Some schools in Estonia permit students to go outside during recess, while only a few have incorporated longer outdoor recess periods (e.g. more than 20 min long) into their schedules. The minimum recess duration for each 45-min academic lesson is 10 min; however, schools are allowed to extend this time. As a result, some schools offer outdoor recesses that last for 30 and 45 min or even longer. The Estonian coalition agreement for 2023–7 commits to expanding existing educational programs that promote PA in all schools, ensuring that students are physically active for at least 60 min during school hours [18]. This highlights the importance of developing effective solutions to enhance students' PA levels during school time.

Therefore, the main aim of this study was to investigate and compare recess and school time PA levels (including all lessons and recesses of the school day) and sedentary behaviour among schools with different recess types among primary school students in Estonia. More specifically, it was investigated how much recess time students spend in MVPA and being sedentary, and what proportion of MVPA minutes of different types of recesses contribute to the total school time MVPA minutes. In addition, it was investigated if the length of recess and different recess types are associated with students’ MVPA and sedentary behaviour.

This study is part of a broader research study that aims to measure students’ daily PA and recess PA levels and to assess how schoolyards with different equipment and affordances are associated with students’ PA levels during outdoor recess [11], as well to explore students’ and their parents’ perceptions about outdoor recess and its benefits, and PA opportunities in schools, which is published elsewhere [19].

## Methods

### Participants

Initially, 19 primary schools in Estonia with an active outdoor or indoor recess were randomly selected from all schools participating in the Schools in Motion program. Out of 19 selected schools, 15 consented to participate in the study. Located throughout Estonia, the schools varied in size and implemented various recess types. All students from grades three to six (9–13-years-olds) were invited to participate in the study. Grades three to six were chosen for this study as children’s PA levels plateau in grade three, followed by a subsequent decline in PA levels and a rise in sedentary behaviour from grades four to six [20]. In total, 967 students wore the accelerometer, whereas 800 students (357 boys, 443 girls; age 10.4 ± 1.3 years) provided valid PA data.

### Instrumentation

The ActiGraph GT3X accelerometer (ActiGraph LLC, Pensacola, FL, USA) was used to monitor PA and sedentary time during waking hours. Participants were asked to wear the accelerometer on the hip for 7 consecutive days except during water-related sports and activities. Although, participants wore the accelerometer during waking hours, as the focus of the study was school time and recess PA, only school time PA was used in the analysis. Valid recording for PA and sedentary time required at least one full school day. The average accelerometer wear time among students included in the analysis was 4.28 ± 0.96 school days. Non-wearing time (at least 20 min of consecutive reading of zero counts and the night-time periods when the unit was removed) was eliminated from the analysis. PA data were analysed using ActiLife software version 6.13.4 (ActiGraph LLC). PA intensity zones and sedentary behaviour were calculated based on Evenson *et al*.’s cut-off points for children [21]. In addition, students recorded daily recess participation in accelerometer diaries for each measurement day. If the student had not marked participation in the outdoor recess or was absent from school, the data for that particular day was excluded from the analysis. Accelerometer diaries provided recess participation data, while school schedules offered by the schools were utilized to extract the recess and school time PA data.

For the statistical analysis, schools were divided into three separate groups based on recess type in schools—(i) ‘daily outdoor recess’; (ii) ‘irregular outdoor recess’; (iii) ‘indoor recess’. Schools in the ‘daily outdoor recess’ group had a daily outdoor recess, where students actively participated (6 schools; *n* = 333 students, 44.2% boys). Lengths of outdoor recesses in different schools in the ‘daily outdoor recess’ group varied between 15 and 50 min and it was mandatory to go outside during this time. Schools in ‘irregular outdoor recess’ group had one active recess every day, on some days of the school week it was an outdoor recess, and on other days it was an active indoor recess where students could play in the gym or engage in other physically active activities (4 schools, *n* = 243 students, 44.9% boys). Recess length in this group varied between 25 and 30 min in different schools. Schools in the ‘indoor recess’ group did not have outdoor recess as part of their school schedule (5 schools; *n* = 224 students, 45.1% boys). Schools in ‘indoor recess’ group had an active indoor recess every day where students could access the gym or engage in other physically active activities. The length of active indoor recesses varied between 15 and 50 min in different schools. The average weekly PA data from outdoor recess was used in the analysis for the ‘daily outdoor recess’ group and weekly average PA data from the active indoor recess was used for the ‘indoor recess’ group. The average PA data from outdoor recess and the equivalent indoor recess data were used for the ‘irregular outdoor recess’ group. In total, 2923 recess data were analysed. 1160, 787, and 976 recesses were analysed in ‘daily outdoor recess’, ‘irregular outdoor recess’, and ‘indoor recess’ groups, respectively.

### Procedure

Data collection occurred from 1 September 2021 to 20 December 2022. Before data collection written informed consent was obtained from all participants and their legal guardians. Members of the research team handed out the accelerometers and participants wore them for the following 7 days. All data handling and analysis were anonymized and in line with ethical guidelines. The study was performed in accordance with the Declaration of Helsinki [22] and was approved by the Medical Ethics Committee of the University of Tartu, Tartu, Estonia, approval no. 330/T-7.

### Data analysis

For statistical analysis SPSS software for Windows (version 29.0) was used. The normality of the data was tested using the Shapiro–Wilk test. A Univariate General Linear Model using Tukey’s *post hoc* test was conducted to examine differences in sedentary behaviour and PA in recess: (i) between school groups; (ii) within sex differences between school groups; (iii) between grades in different school groups. The analyses were adjusted for the length of the recess (except when % values were used), sex (except analyse 2, where sex was used as fixed factor), and grade (except analyse 3, where the grade was used as a fixed factor). Statistical significance was set at *P* < .05.

## Results

The average time students spent in different PA intensities during recess is presented in Table 1. ‘Indoor recess’ group spent significantly more recess time in sedentary and significantly less time in light PA and MVPA, compared to ‘daily outdoor recess’ and ‘irregular outdoor recess’ groups (*P* < .05). At the same time ‘irregular outdoor recess’ group had significantly higher sedentary behaviour compared to the ‘daily outdoor recess’ group (*P* < .05). 13.5% of students in the ‘daily outdoor recess’ group, 7.1% of students in the ‘indoor recess’ group, and 8.2% of students in the ‘irregular outdoor recess’ group spent at least 40% of recess time in MVPA. In the ‘daily outdoor recess’ and ‘irregular outdoor recess’ groups, respectively, 29.1% and 25.5% of students spent at least 30% of recess time in MVPA. The same indicator for the ‘indoor recess’ group was only 14.3% of students.

**Table 1.** Differences in time spent in different activity levels (mean ± SE) during recess in school groups

	Daily outdoor recess (*n* = 333)		Irregular outdoor recess (*n* = 243)		Indoor recess (*n* = 224)	
	Min/recess^a^	%^b^	Min/recess^a^	%^b^	Min/recess^a^	%^b^
Sedentary behaviour	8.7 ± 0.3	30.8 ± 0.8	10.3 ± 0.3^c^	34.0 ± 1.0^c^	13.1 ± 0.3^c^^,^^d^	43.6 ± 1.0^c^^,^^d^
Light PA	13.3 ± 0.2	45.4 ± 0.6	12.9 ± 0.2	44.5 ± 0.7	11.7 ± 0.2^c^^,^^d^	40.4 ± 0.7^c^^,^^d^
MVPA	7.2 ± 0.2	23.8 ± 0.7	6.0 ± 0.2^c^	21.5 ± 0.8	4.3 ± 0.2^c^^,^^d^	15.8 ± 0.8^c^^,^^d^

a Adjusted for the length of outdoor recess, sex, and grade.

b Adjusted for sex and grade.

c Significantly different from the ‘daily outdoor recess’ group.

d Significantly different from the ‘irregular outdoor recess’ group.

Table 2 compares the time spent in sedentary behaviour and PA in recess within sex differences between school groups. Both, boys, and girls in the ‘indoor recess’ group engaged more in sedentary behaviour and less MVPA compared to boys and girls in the ‘daily outdoor recess’ group (*P* < .05). Within girls, the ‘daily outdoor recess’ group and ‘irregular outdoor recess’ group had significantly higher PA compared to the ‘indoor recess’ group (*P* < .05).

**Table 2.** Differences in time spent in different activity levels (mean ± SE) during recess within sexes in school groups

	Boys (*n* = 357)			Girls (*n* = 443)		
	Daily outdoor recess (*n* = 147)	Irregular outdoor recess (*n* = 109)	Indoor recess (*n* = 101)	Daily outdoor recess (*n* = 186)	Irregular outdoor recess (*n* = 134)	Indoor recess (*n* = 123)
Sedentary behaviour (%)	27.9 ± 1.3	30.7 ± 1.5	37.7 ± 1.5^a^^,^^b^	33.2 ± 1.1^c^	36.6 ± 1.3^c^	48.3 ± 1.4^a^^,^^b^^,^^c^
Light PA (%)	45.3 ± 0.9	45.0 ± 1.0	41.6 ± 1.0^a^	45.5 ± 0.8	44.1 ± 0.9	39.5 ± 0.9^a^^,^^b^
MVPA (%)	26.8 ± 1.0	24.3 ± 1.2	20.6 ± 1.2^a^	21.3 ± 0.9^c^	19.3 ± 1.0^c^	11.9 ± 1.1^a^^,^^b^^,^^c^

Adjusted for the grade.

a Significantly different from the ‘daily outdoor recess’ group.

b Significantly different from the ‘irregular outdoor recess’ group.

c Significantly different from boys.

Boys in all three groups spent more time in MVPA and less time sedentary compared to girls (Table 2). Significant differences between boys and girls within all three groups were found for MVPA and sedentary behaviour (*P* < .05).

**Figure 1.**
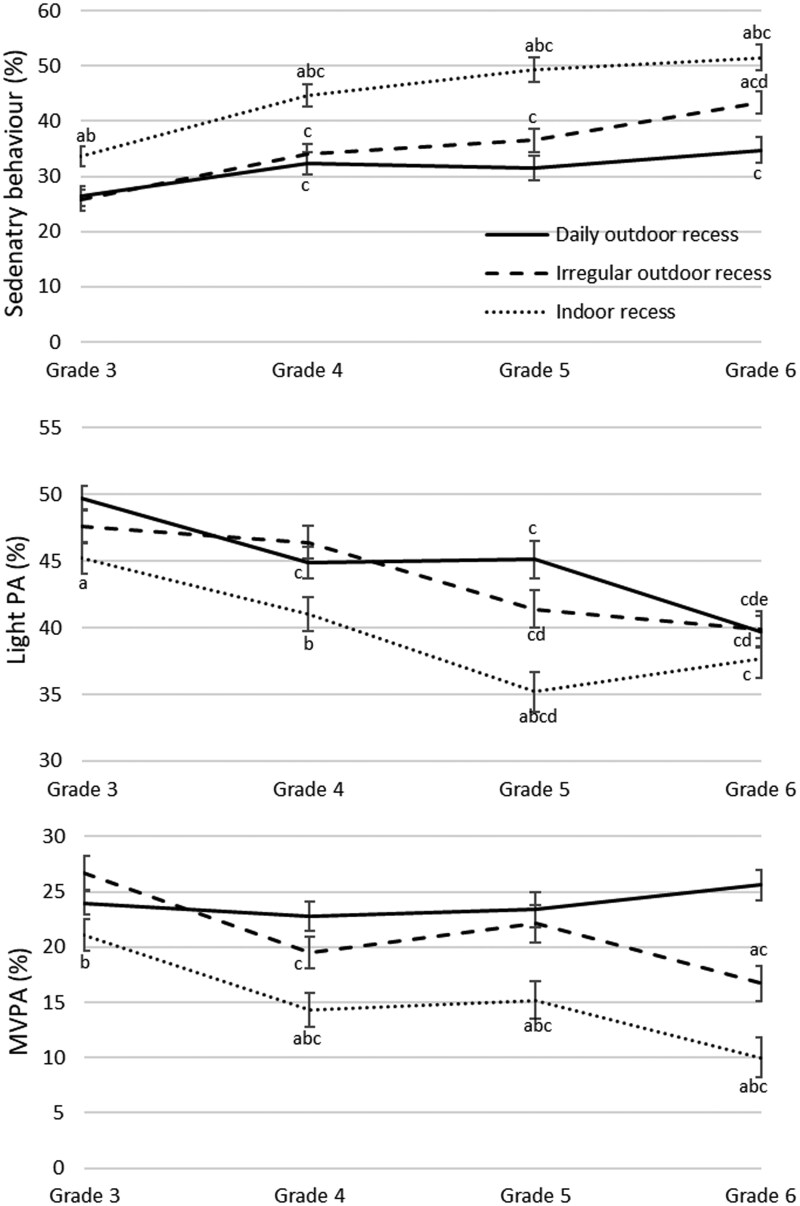
Differences in time spent in different activity levels during recess in grades 3–6. (a) Significantly different from ‘Daily outdoor recess’; (b) significantly different from ‘Irregular outdoor recess’; (c) significantly different from grade 3; (d) significantly different from grade 4; (e) significantly different from grade 5.

Figure 1 compares time spent in sedentary behaviour and PA in a recess within grades three to six between school groups. The ‘indoor recess’ group had significantly higher sedentary behaviour and lower MVPA, compared to the ‘daily outdoor recess’ group (except for MVPA in grade 3) and the ‘irregular outdoor recess’ group in all grades (*P* < .05). Results also indicated the trend for increased sedentary time and decreased MVPA with age. Sedentary time was significantly lower in grade 3 than in other grades in all school groups (*P* < .05), except for grade 5 in the ‘daily outdoor recess’ group. However, the most notable change in sedentary behaviour was seen in the ‘indoor recess’ group, where students in grade 3 spent significantly less recess time sedentary compared to grade 6 (*P* < .05). MVPA was significantly higher in grade 3 for ‘indoor recess’ and ‘irregular outdoor recess’ groups compared with other grades, except for grade 5 in ‘irregular outdoor recess’ group (*P* < .05). However, time spent in MVPA for the ‘daily outdoor recess’ group remained similar for all grades (22.8%–25.6%), and no significant difference was found.

The average time students spent in different PA intensities during recesses in varying lengths are presented in Table 3. The ‘daily outdoor recess’ group spent significantly less time in sedentary and more time in MVPA compared to the ‘indoor recess only’ group during all recess lengths (*P* < .05), except for 30 min recess. Statistical significances were also found for all PA intensities during 25 min recess between the ‘indoor recess only’ and ‘irregular outdoor recess’ groups and for MVPA and sedentary behaviour during 40–50 min recess between ‘daily outdoor recess’ group and ‘indoor recess’ group (*P* < .05).

**Table 3.** Differences in time spent (time in minutes and amount in %) in different activity levels (mean ± SE) during recesses of varying length in school groups

	15–20 min recess			25 min recess			30 min recess			40–50 min recess		
	Daily outdoor recess	Irregular outdoor recess	Indoor recess	Daily outdoor recess	Irregular outdoor recess	Indoor recess	Daily outdoor recess	Irregular outdoor recess	Indoor recess	Daily outdoor recess	Irregular outdoor recess	Indoor recess
Sedentary behaviour (min/recess)	2.9 ± 1.4 (17%)	–	7.8 ± 0.5 (44%)^a^	6.1 ± 0.5 (24%)	7.7 ± 0.4 (31%)^a^	10.9 ± 0.6 (44%)^a^^,^^b^	9.5 ± 0.4 (31%)	11.1 ± 0.4 (37%)^a^	10.2 ± 0.6 (34%)	15.2 ± 0.5 (37%)	–	21.5 ± 0.7 (54%)^a^
Light PA (min/recess)	11.0 ± 1.0 (52.5%)	–	7.2 ± 0.4 (40%)^a^	11.6 ± 0.4 (48%)	11.5 ± 0.3 (46%)	10.4 ± 0.4 (41%^c^^,^^d^)	13.8 ± 0.3 (46%)	13.0 ± 0.3 (43%)	12.4 ± 0.5 (42%)^a^	17.8 ± 0.3 (42%)	–	15.3 ± 0.5 (38%)^a^
MVPA (min/recess)	6.3 ± 1.1 (30.5%)	–	2.6 ± 0.4 (16%)^a^	7.1 ± 0.4 (28%)	5.8 ± 0.3 (23%)^a^	3.6 ± 0.5 (15%)^a^^,^^b^	6.6 ± 0.3 (23%)	5.9 ± 0.3 (20%)	7.2 ± 0.5 (24%)	9.0 ± 0.4 (21%)	–	3.2 ± 0.5 (8%)^a^

Adjusted for the grade and sex. Schools with outdoor recess on some days did not have recesses with a length of 15–20 min and 40–50 min.

a Time in minutes and amount in % significantly different from the ‘daily outdoor recess’ group.

b Time in minutes and amount in % significantly different from the ‘irregular outdoor recess’ group.

c Amount in % significantly different from the ‘daily outdoor recess’ group.

d Amount in % significantly different from the ‘irregular outdoor recess’ group.

‘Indoor recess’ group accumulated significantly less total school time MVPA minutes (22.8 ± 10.6 min) compared to ‘daily outdoor recess’ (27.0 ± 10.6 min) and ‘irregular outdoor recess’ group (24.7 ± 9.5 min) (*P* < .05). Recess MVPA minutes in ‘daily outdoor recess’ (27.7 ± 0.8%) group contributed significantly more total school time MVPA, compared to ‘irregular outdoor recess’ (24.5 ± 0.9%) group and ‘indoor recess’ (17.8 ± 0.9%) group (*P* < .05). Girls in the ‘daily outdoor recess’ group acquired 28.3 ± 1.0% of total school time MVPA minutes during outdoor recess compared to boys 26.9 ± 1.1%; however, no significant difference was found. Within sex both, boys and girls in ‘daily outdoor recess’ and ‘irregular outdoor recess’ (boys 24.6 ± 1.3% and girls 24.4 ± 1.2%) groups acquired more total school time MVPA during recess compared to the ‘indoor recess’ (boys 19.8 ± 1.4% and girls 16.2 ± 1.2%) group (*P* < .05).

## Discussion

The main aim of the study was to investigate and compare recess and school time PA levels (including all lessons and recesses) and sedentary behaviour in Estonian primary schools with different recess types. Accelerometer data revealed that students in the ‘daily outdoor recess’ group had higher MVPA levels and lower sedentary behaviour during recess, and recess time MVPA did not decrease with increasing age in the ‘daily outdoor recess’ group. Moreover, irregular outdoor recess was associated with higher PA levels, and even 15–20 min recess contributed significantly towards students’ school time MVPA.

In this study, students in the ‘irregular outdoor recess’ group obtained significantly less MVPA during recess compared to the students in the ‘daily outdoor recess’ group but still accumulated significantly more MVPA compared to the ‘indoor recess’ group. Furthermore, students in the ‘daily outdoor recess’ and ‘irregular outdoor recess’ groups accumulated 27.7% and 24.5% of total school time MVPA during recess, respectively. While students in the ‘indoor recess’ group obtained only 17.5% of total school time MVPA during recess. Erwin *et al*. found that students accumulated approximately one-third of total school time PA during unstructured recess [6], which was similar to the results of ‘daily outdoor recess’ and ‘irregular outdoor recess’ groups in this study. These findings highlight that even occasional outdoor recess contributes to higher PA levels. Dessing *et al*. further emphasize the positive impact of schoolyards, particularly during recess, on MVPA [23]. Therefore, providing an unstructured outdoor recess is crucial for promoting students’ PA, and fostering diverse play, skill development, mental breaks, and social interaction [24].

PA tends to decline with age due to decreased interest and time constraints of students’ day-to-day activities [25]. In this study, even though sedentary time increased for the ‘daily outdoor recess’ group with age, there was no significant difference in time spent in MVPA in all grades (22.8%–25.6%). In addition, students within all grades in ‘daily outdoor recess’ and ‘irregular outdoor recess’ groups spent significantly less recess time sedentary and significantly more recess time in MVPA, except for third grade in the ‘daily outdoor recess’ group, compared to ‘indoor recess’ group. Even with increased sedentary time, older students’ MVPA during outdoor recess remained significant, highlighting the value of outdoor recess in maintaining PA levels as students get older.

In this study, boys in the ‘daily outdoor recess’ group spent 26.8% of the recess time in MVPA compared to 21.3% for girls in the same school group. Boys are generally more active than girls, often engaging in competitive play, while girls prioritize social interaction [11, 26]. Other studies confirm these findings, with boys spending 32.9%–39.5% and girls spending 23.4%–25.3% of recess time in MVPA [23, 27, 28]. However, boys and girls in this study in general spent less recess time in MVPA compared to the results of the previous studies [23, 27, 28]. Compared to the boys and girls in this study, Saint-Maurice *et al*. found that boys and girls spent more than twice as much recess time engaged in MVPA, 69.2% for boys and 52.3% for girls, respectively [29]. Both boys and girls in this study, with girls slightly exceeding boys, obtained ∼30% of their school time MVPA during outdoor recess, although the difference was not statistically significant.

Despite research on recess PA [9, 10], no official recommendation of the most influential recess length is established. Erwin *et al*. found that even a 15 min recess affects students’ total school day PA [6]. Similarly, in this study, students showed the highest MVPA and lowest sedentary behaviour during a 15–20 min outdoor recess in all school groups. This supports Erwin *et al*. finding that recess length does not have to be extremely long to significantly affect students’ PA accumulation [6] and even a short 15–20 min outdoor recess can be a good addition to the school day to support students’ PA accumulation. In this study, the percentage of recess MVPA time decreased, and time spent in sedentary behaviour increased with longer recess length, however, longer recess time (e.g. recess length of 30–50 min) allowed higher MVPA minutes accumulation. Besides increased MVPA minutes, longer recesses offer additional benefits such as social interaction, cognitive restoration, and improved focus [7, 8]. In addition, the proportion of light PA remained relatively high for all recess lengths in all three groups. This implies that during shorter recess (e.g. 15–20 min recess time), students are motivated to play more moderately and vigorously whereas during longer recesses the proportion of sedentary behaviour increases. Activities during longer recess can be more varied and it also provides opportunities for light PA activities. While MVPA has the most known health benefits, light PA also contributes to cognitive development, mental well-being, and physical literacy in children [11, 24].

Research suggests that students should spend at least 40%–50% of recess time in MVPA [6, 12, 13]. The average amount of recess time MVPA in all three groups (23.8%–15.8%) was considerably lower than the suggested recommendation. Only 13.5% of students in the ‘daily outdoor recess’ group met the suggested 40% recess PA recommendation. The same indicator for the other two groups was even lower. Ridgers *et al*. found similar results with 14.9% of the boys and 4.3% of the girls meeting the 40% recommendation [28], whereas Tercedora *et al*. found that 25.4% of students in their study met the suggested recommendation [27]. As outdoor recess in Estonia is a fairly new addition to the school schedule, spending 40%–50% of recess time in MVPA might seem disproportionate. 29.1% of students in ‘daily outdoor recess’ and 25.5% of students in ‘irregular outdoor recess’ groups spent at least 30% of the longest recess in MVPA. Significantly more students spent at least 30% of recess time in MVPA, which makes a 30% recommendation more plausible in the Estonian context.

### Limitations

Strengths of this study include its large sample size, the number of schools that participated in the study, and objective accelerometer-measured PA. Additionally, almost 3000 recesses were analysed during statistical analysis, and it was the first study to investigate the PA levels during recess in Estonia in complex. Outdoor recess is not a common part of the daily school schedule in Estonia, and it can be affected by weather conditions and the schoolyard design. A potential limitation is the variability in seasonal conditions during PA data collection across schools. As temperature can significantly impact MVPA during outdoor play; future studies should standardize data collection to one season for more objective PA data. However, in this study, in schools where outdoor recess was mandatory for everybody, students were used to going outside despite the weather conditions, but it is still possible that weather conditions affected children’s PA to some extent. In addition, a previous study found that among schools in Estonia, natural and spacious schoolyards generated more PA during outdoor recess compared to smaller and artificial schoolyards [11]. As all schoolyards in this study are different in size and have various possibilities for PA, it is likely that the PA levels of students might have also been affected by the schoolyard design. Therefore, future research should also consider schoolyard design when measuring outdoor recess PA levels.

## Conclusions

In conclusion, the results of this study emphasized the positive association between outdoor recess and students’ PA. Daily outdoor recess supported students’ PA the most. However, irregular outdoor recess also contributed more towards students’ PA compared to schools with only indoor recess. In addition, it was found that MVPA stayed similar for the ‘daily outdoor recess’ group with an increasing age. Therefore, it is an important finding for helping to maintain MVPA levels as children grow. While longer 40–50 min recesses promote students’ cognitive and mental health due to the dominance of light activities, in this study significant levels of MVPA were already achieved within shorter 15–20 min recesses. These findings suggest that strategically incorporating outdoor recess into the school schedule can significantly enhance students’ MVPA levels, as well as their cognitive and mental well-being. Furthermore, even a shorter irregular outdoor recess yields greater benefits than having only indoor recess.

Conflict of interest: None declared.

## Funding

This study was supported by the project ‘Increasing Physical Activity of Schoolchildren’ funded by EEA grants (grant 2014-2021.1.05.20-0004) under the programme ‘Local Development and Poverty Reduction’, co-financed by the Ministry of Social Affairs and the University of Tartu.

## Data Availability

The datasets presented in this article are not readily available because data cannot be shared publicly as our participants and their legal guardians were not asked to consent to data-sharing outside of our research group. Availability of data has to be in accordance with the Medical Ethics Committee of the University of Tartu, Tartu, Estonia. Requests to access the datasets should be directed to Evelin Mäestu, evelin.maestu@ut.ee. Key points MVPA levels during outdoor recess did not significantly change among students in the ‘daily outdoor recess’ group with increasing age, highlighting that daily outdoor recess helps to prevent a decrease in PA during recess with increasing age. Students in the ‘irregular outdoor recess’ group obtained higher PA levels and lower sedentary time during recess compared to the students in the ‘indoor recess’ group, emphasizing that even an irregular outdoor recess is associated with higher PA levels of students. During a 15–20 min outdoor recess students spent the most amount of recess time in MVPA compared to longer recesses, however, a longer recess of 40–50 min is important for students’ cognitive and mental health due to the dominance of light activities.
